# Bimodal Therapy for Chronic Subjective Tinnitus: A Randomized Controlled Trial of EMDR and TRT Versus CBT and TRT

**DOI:** 10.3389/fpsyg.2020.02048

**Published:** 2020-09-10

**Authors:** Tine Roanna Luyten, Laure Jacquemin, Nancy Van Looveren, Frank Declau, Erik Fransen, Emilie Cardon, Marc De Bodt, Vedat Topsakal, Paul Van de Heyning, Vincent Van Rompaey, Annick Gilles

**Affiliations:** ^1^Faculty of Medicine and Health Sciences, Department Translational Neuroscience, University of Antwerp, Antwerp, Belgium; ^2^Department of Otorhinolaryngology and Head and Neck Surgery, Antwerp University Hospital, Edegem, Belgium; ^3^Hoorzorg Van Looveren BVBA, Borsbeek, Belgium; ^4^Faculty of Education, Health and Social Work, University College Ghent, Ghent, Belgium

**Keywords:** bimodal therapy, chronic subjective tinnitus, tinnitus retraining therapy, cognitive behavioral therapy, eye movement desensitization and reprocessing, psychotherapy

## Abstract

**Introduction:**

To date, guidelines recommend the use of a stepped care approach to treat tinnitus. The current clinical management of tinnitus frequently consists of audiologic interventions and tinnitus retraining therapy (TRT) or cognitive behavioral therapy (CBT). Due to the high heterogeneity of the tinnitus population and comorbidity of tinnitus with insomnia, anxiety, and depression, these interventions may not be sufficient for every patient. The current study aims to determine whether a bimodal therapy for chronic, subjective tinnitus consisting of the combination of TRT and eye movement desensitization reprocessing (EMDR) results in a clinically significant different efficacy in comparison with the prevailing bimodal TRT and CBT therapy.

**Methods:**

Patients were randomized in two treatment groups. The experimental group received the bimodal therapy TRT/EMDR and the active control group received the bimodal therapy TRT/CBT. Evaluations took place at baseline (T_0_), at the end of the treatment (T_1_), and 3 months after therapy (T_2_). The tinnitus functional index (TFI) was used as primary outcome measurement. Secondary outcome measurements were the visual analog scale of tinnitus loudness (VAS_Loudness_), tinnitus questionnaire (TQ), hospital anxiety and depression scale (HADS), hyperacusis questionnaire (HQ), global perceived effect (GPE), and psychoacoustic measurements.

**Findings:**

The TFI showed clinically significant improvement in both bimodal therapies (mean decrease 15.1 in TRT/CBT; *p* < 0.001 vs. 16.2 in TRT/EMDR; *p* < 0.001). The total score on the TQ, HADS, HQ, and VAS_Loudness_ all demonstrated significant decrease after treatment and follow-up (*p* < 0.001) in the experimental and the active control group. GPE-measurements revealed that more than 80% (i.e., 84% in TRT/CBT vs. 80% in TRT/EMDR) of the patients experienced substantial improvement of tinnitus at follow up. Treatment outcome remained stable after 3 month follow-up and no adverse events were observed.

**Conclusion:**

Both psychotherapeutic protocols result in a clinically significant improvement for patients with chronic subjective tinnitus. No significant different efficacy was found for the TRT/EMDR treatment compared to the combination of TRT and CBT.

**Clinical Trial registration:**

ClinicalTrials.gov, ID: NCT03114878. April 14, 2017.

## Introduction

Tinnitus, the perception of sound in the absence of an external sound source, also known as phantom percept (Baguley et al., 2013), can be extremely bothersome. About 8–20% of the population report chronic tinnitus and for 1–3% of these patients the sound is so debilitating that professional help is necessitated (Heller, 2003). Tinnitus can be experienced as stressful, irritating, frightening, alarming and can have a huge impact on quality of life. Recent findings show high comorbidity with psychological and psychosomatic symptoms and/or psychiatric syndromes such as anxiety disorders, insomnia, and depression (Eysel-Gosepath and Selivanova, 2005; Malakouti et al., 2011; Geocze et al., 2013; Pinto et al., 2014; Pattyn et al., 2016). The tinnitus population is highly heterogeneous, often requiring a combination of therapeutic support to manage the complexity of the symptoms. Hence, a specialized and client-centered treatment plan executed by a multidisciplinary team of professionals is recommended (Van de Heyning et al., 2015).

Clinical management with the integration of audiological and psychological interventions such as tinnitus retraining therapy (TRT) and cognitive behavioral therapy (CBT) is regularly introduced (Langguth et al., 2013; Shi et al., 2014). TRT management consists of reassurance, education, and use of sound enrichment through hearing aids or sound generators in order to reduce the tinnitus percept. Caregivers often use some form of TRT (Phillips and McFerran, 2010) or offer counseling based on the neurophysiological model of Jastreboff and Jastreboff (2000) and Jastreboff P. J. (2007). CBT as a treatment for tinnitus focuses on modifying dysfunctional thoughts and coping strategies in order to change the emotional reactions toward the tinnitus sound (Martinez-Devesa et al., 2007; Hesser et al., 2011). TRT and CBT may lead to habituation of the tinnitus sound, helpful thoughts, and adapted reactions toward the sound. Different treatment protocols were developed ranging from an intensive CBT-approach (Aazh et al., 2016; Aazh and Moore, 2018) to a stepped-care-approach (Cima et al., 2012, 2014). To date, the European guidelines strongly recommend CBT to treat tinnitus (Cima et al., 2019). Nevertheless, in some cases extensive CBT and/or TRT programs do not render sufficient tinnitus relief for patients, thus additional therapies may be required.

Chronic, subjective tinnitus is considered a medically unexplained symptom as, to date, there is no consensus concerning the underlying pathophysiology (Hiller et al., 1997, 1999). Eye movement desensitization reprocessing (EMDR) has proven to be a highly effective treatment for medically unexplained symptoms (Wilensky, 2006; Van Rood and De Roos, 2009; de Roos et al., 2010; Iranshahr, 2014; Rostaminejad et al., 2017) such as chronic pain, chronic headache, and chronic fatigue syndrome. The mechanisms that underpin EMDR are not yet fully understood. This well-structured psychotherapeutic method is based on the theoretical model called “adaptive information processing (AIP)” (Shapiro, 2001; Solomon and Shapiro, 2008). AIP is the capacity of the human brain to process experiences to develop an adaptive solution for the context one is living in. Trauma can obstruct this natural information processing system and therefore specific methods are necessary to facilitate this innate ability to adapt (Shapiro, 2007). At the level of the auditory regions of the brain, tinnitus can be perceived as trauma. The emotional reactions associated with the tinnitus sound trigger networks at the level of the insula, hippocampus, parahippocampus, anterior cingulate cortex and the amygdala. A tangle of auditory and non-auditory networks influence each other when tinnitus becomes persistent and chronic (Mirz et al., 2000; Schlee et al., 2009; Vanneste et al., 2010; De Ridder et al., 2011). There is a need to understand the relationship between EMDR as a possible facilitator and the processing of tinnitus. Previous research has shown that the bilateral stimulation used in EMDR increases limbic processing together with decreasing frontal activation. Integration and reintegration of information about the tinnitus could therefore be facilitated through these patterns of neural activity. New adaptive reactions to the tinnitus sound could lead to new neural networks causing the tinnitus sound to be processed as a non-threatening and neutral sound (Herkt et al., 2014; Boukezzi et al., 2017).

Few studies have reported the use of EMDR as a treatment method for tinnitus (Zengin, 2009; Hochstenbach-Nederpel, 2015; Rikkert et al., 2018; Phillips et al., 2019), but outcomes remain unclear. Although some research has been carried out on EMDR and tinnitus, only two studies have attempted to investigate the efficacy systematically. Rikkert et al. (2018) conducted a pilot study showing promising results in treating 35 participants with high levels of chronic tinnitus distress with EMDR. Compared to the waiting list condition, scores on the tinnitus functional index (TFI), mini-tinnitus questionnaire (Mini-TQ), and symptom checklist-90 (SCL-90) decreased significantly in the treatment group (Rikkert et al., 2018). A recent pilot study with a sample size of 14 participants was published, reporting a decrease of more than 20 points on the tinnitus handicap inventory (THI) after EMDR treatment (Phillips et al., 2019). However, the effects of EMDR therapy in tinnitus remain elusive and further research is required (Luyten et al., 2020).

This prospective study was designed to examine the effect of EMDR as possible psychotherapeutic treatment for chronic, subjective tinnitus. A randomized controlled trial was set up to investigate (1) whether a bimodal therapy containing of a therapy protocol of TRT and EMDR results in a clinically significant different efficacy compared to the standard treatment combining TRT and CBT and (2) to evaluate the effect of the bimodal treatments on the primary and secondary outcome measurements. To our knowledge, this is the first RCT that explores both therapeutic interventions simultaneously.

## Materials and Methods

### Study Design

**FIGURE 1:**
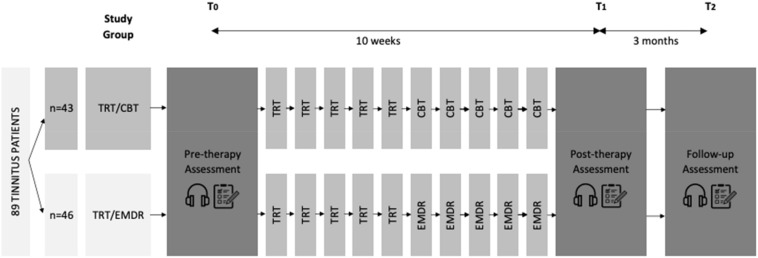
Study design. A total of 89 participants were divided into two treatment groups: TRT/CBT (*n* = 43) or TRT/EMDR (*n* = 46). Each group received five sessions of TRT and five sessions of either CBT or EMDR, with one session each week. Psychoacoustic measurements 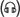 and questionnaires 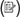 were part of the assessment performed at pre-therapy (T_0_), post-therapy (T_1_), and follow-up (T_2_) (CBT, cognitive behavioral therapy; EMDR, eye movement desensitization and reprocessing; TRT, tinnitus retraining therapy).

This prospective, randomized, controlled clinical trial with blind evaluator was conducted at the University Hospital of Antwerp and the multidisciplinary private practice Hoorzorg Van Looveren in Borsbeek, Belgium. All participants were randomized in one of the two treatment groups: one group received a combination of TRT and CBT while the other group received a combination of TRT and EMDR. Patients were given 60 min TRT sessions for five consecutive weeks, 60 min psychotherapy sessions (CBT or EMDR) for five consecutive weeks and received a 60 min follow-up session 3 months after the last psychotherapy session. Because of the intensive therapy trajectory patients were followed up closely from the inclusion until 9 months later. Figure 1 displays the study design that was applied.

All patients signed an informed consent form prior to enrollment. The Ethical Committee of the Antwerp University Hospital approved the study protocol on October 17, 2016 with protocol number EC UZA 16/35/360. This study is registered at ClinicalTrials.gov, number NCT03114878.

### Randomization and Masking

Patients were referred by the Ear, Nose, and Throat (ENT) department of the Antwerp University Hospital and were randomized in the active control group (TRT/CBT) or the experimental group (TRT/EMDR) by use of a stratification paradigm according to TFI grade (exclusion of grade 1). The allocation sequence was determined by the date that patients were referred to the study. Patients were masked to the treatment group allocation. Participants were not aware of the alternative treatment to prevent bias and influencing the motivation of the patients. The investigators, as well as the TRT-therapist, were also masked to the treatment group. The psychotherapist who conducted the CBT and the EMDR treatment was not blinded and informed the participants about the study protocol.

### Participants

**FIGURE 2:**
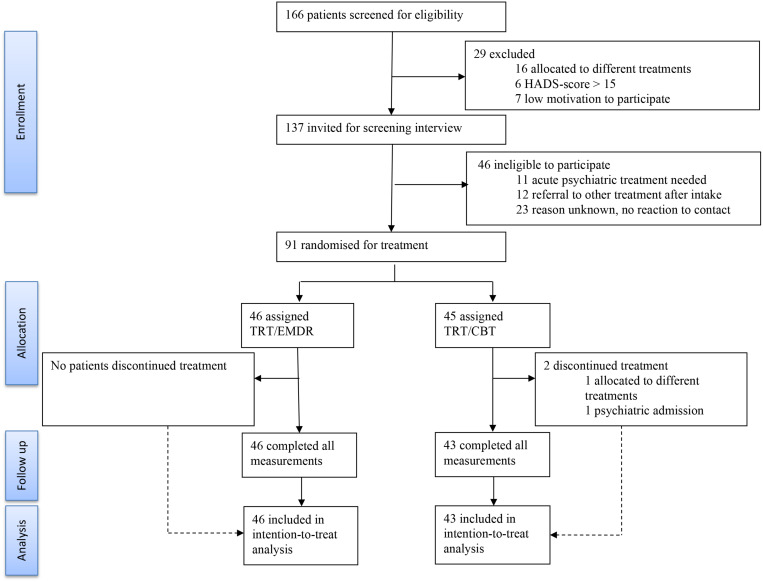
Consort flow diagram. Trial profile of the 166 patients who were screened for eligibility. A total of 89 patients completed the study. (TRT, tinnitus retraining therapy; CBT, cognitive behavioral therapy; EMDR, eye movement desensitization and reprocessing; HADS, hospital anxiety and depression scale).

Patients aged 18–75 years old with chronic, subjective, non-pulsatile tinnitus (>3 months) (Cederroth et al., 2019) were eligible for inclusion. A score of 25 or more, but no higher than 90, on the TFI and stable use of medication during the study was considered necessary to take part. An anxiety and depression subscale score on the hospital anxiety and depression scale (HADS) of more than 15 and a score of more than 40 on the hyperacusis questionnaire (HQ) were used as cut-off scores for a referral to other treatments. The diagnosis of an objective, pulsatile tinnitus, an active middle ear pathology, neurological and psychiatric co-morbidity for which acute psychotherapy was ongoing, psychosis, schizophrenia, epilepsy, or pregnancy also lead to exclusion of the study. From October 2016 to April 2019 137 patients were randomized from a total of 166 patients who were screened at the University Hospital of Antwerp. A total of 91 patients started the treatment and 89 patients completed the therapeutic trajectory. Figure 2 shows the trial profile, including the reasons for exclusion and drop-out. Only two patients discontinued treatment because of intrusive reasons, more specifically one patient was admitted to a psychiatric facility for complaints other than tinnitus and one patient did not respect the treatment protocol by initiating other treatments (i.e., neuromodulation, medication, and infiltration).

The baseline characteristics of the participants are presented in Table 1. The audiometric baseline evaluation consisted of pure tone audiometry and tympanometry. The pure tone audiometry was conducted using a two-channel Interacoustics AC-40 audiometer (Interacoustics A/S, Middelfart, Denmark) in a sound-treated booth. Air conduction thresholds were determined using a TDH-39 headphone at frequencies from 125 to 16 kHz (International Organization for Standardization (ISO) 8253–1, 2010). In addition, active middle ear pathology could be excluded by performing tympanometry. The two groups (i.e., TRT/EMDR vs. TRT/CBT) did not differ significantly in these baseline characteristics (*p* > 0.05).

**TABLE 1:** Baseline characteristics.

Characteristics	Total	TRT/EMDR	TRT/CBT
Number of subjects	89	46	43
Age	47.87 (12.67)	47.52 (12.25)	48.23 (13.39)
**Sex**			
Male	63(70.8%)	30(65.2%)	33(76.7%)
Female	26(29.2%)	16(34.8%)	10(23.3%)
Mean duration of tinnitus	7 years (8.66)	7 years (7.68)	8 years (9.66)
**Tinnitus sound**			
Pure tone	56(62.9%)	29(63%)	27(62.8%)
Noise	22(24.7%)	11(23.9%)	11(25.6%)
Polyphonic	11 (12.4)	6(13%)	5(11.6%)
**Tinnitus side**			
Left	21(23.6%)	10(21.7%)	11(25.6%)
Right	7(7.9%)	5(10.9%)	2(4.7%)
Bilateral	39(43.8%)	18 (39.1)	21(48.8%)
Central	22(24.7%)	13(28.3%)	9(20.9%)
PTA left ear	18.22 (17.01)	16.93 (16.51)	19.17 (17.60)
PTA right ear	15.50 (14.06)	15.37 (14.36)	15.54 (13.57)
TFI	51.81 (20.18)	53.88 (18.40)	49.60 (21.93)
TQ	40.40 (16.04)	39.65 (15.19)	41.21 (17.04)
HQ	22.72 (8.13)	22.98 (7.29)	22.44 (9.02)
**HADS**			
Anxiety	9.44 (4.31)	9.39 (4.11)	9.49 (4.57)
Depression	7.40 (4.20)	7.54 (4.38)	7.26 (4.03)
VAS_Loudness_	60.03 (22.68)	60.49 (23.90)	59.55 (21.57)

*Data are mean (SD) or n (%), SD, standard deviation; PTA, pure tone average for 1, 2, and 4 kHz; TFI, tinnitus functional index; TQ, tinnitus questionnaire; HQ, hyperacusis questionnaire; HADS, hospital anxiety and depression scale; VAS_*Loudness*_, Visual Analog Scale for tinnitus loudness.*

### Procedures

Licensed therapists carried out all treatments. One certified therapist performed the modified TRT protocol. Another certified psychotherapist conducted the EMDR and CBT to exclude the bias of the therapist effect. The variable of location could also be controlled by conducting all the therapy sessions in the private practice. All patients received the same amount of contact hours during the treatment. After an initial audiological intake at the University Hospital of Antwerp, patients were referred to the psychotherapist for the screening interview. During this intake session the patient history, complaints associated with the tinnitus, possible comorbidities, important life events, motivation, and availability were queried. After this interview patients were randomized in one of the two bimodal therapies: TRT/CBT or TRT/EMDR. The complete study protocol is described in Luyten et al. (2019).

### Tinnitus Retraining Therapy

Both bimodal therapies were designed with the inclusion of TRT as a large amount of the referred patients received some form of education about the auditory system and the use of sound enrichment before entering the study. To ensure that all patients started from the same level of psychoeducation, we chose to implement TRT as this educational-based treatment consists of a protocollary approach feasible to replicate by therapists enhancing validity and reliability and the proven effectivity of the treatment (Jastreboff P. J., 2007; Grewal et al., 2014; Van de Heyning et al., 2015). The general principles and methodology described by Prof. Pawel Jastreboff and Dr. Jonathan Hazell were carried out (Jastreboff and Jastreboff, 2000; Jastreboff M. M., 2007). This tinnitus-specific educational counseling focuses on how to deal with the emotional and physical responses of tinnitus. The patient counseling guideline of Henry et al. (2007) was used to help the patient habituate to the bothersome sound. The main focus and content of TRT were respected. The components that were highlighted are shown in Appendix 1.

### Cognitive Behavioral Therapy

Originally CBT is based on the assumption that thoughts influence emotions and behaviors (Butler et al., 2006; Wright, 2006). Thus, if thoughts about the tinnitus change, the emotional and behavioral response can change as a consequence. During therapy, the main focus was cognitive restructuring, the perception of the tinnitus and the experiences of the patient. The CBT that was implemented was conducted by a certified psychotherapist. Supervision was organized by a registered CBT-therapist. Appendix 1 demonstrates the content of the CBT-sessions.

### Eye Movement Desensitization and Reprocessing

Eye movement desensitization and reprocessing was developed by Francine Shapiro in 1987. This psychotherapeutic method consists of an eight-phase protocol. This protocol was respected as the therapeutic sessions were conducted following the guidelines created by Shapiro (2001). Bilateral stimulation was utilized to activate the left and right hemisphere during the desensitization phase and induce taxing of the working memory. All patients started with the alternating visual stimuli as this form of stimulation tends to provide the best evidence for effectiveness (Maxfield, 2008; van den Hout et al., 2011). In some cases the tactile stimuli were induced and only one patient tried the headphones elicitating auditive side to side stimuli. The Eyescan 4000 was employed to generate the eye movements, and tactile or auditory stimuli to activate the left and right side of the brain (Shapiro, 2007). The use of this device was chosen to create the opportunity to count and compare the total number of stimuli used and to guarantee test-retest reliability. The psychotherapist was a licensed clinical psychologist, a Europe Practitioner in EMDR and received supervision from a registered EMDR supervisor. The combination of standardized procedures performed during bimodal therapy 2 is summarized in Appendix 2.

### Outcomes

#### Primary Outcome Measurement

##### Tinnitus functional index

All measurements were assessed at baseline before therapy (T_0_), within 1 week after therapy (T_1_) and 3 months after the last therapy session (T_2_). The primary outcome was the TFI. The eight main domains that can be affected by tinnitus (intrusiveness, reduced sense of control, cognitive interference, sleep disturbance, auditory difficulties attributed to tinnitus, interference with relaxation, reduced quality of life and emotional distress) were investigated through this questionnaire. This self-reported questionnaire consists of 25 questions with Likert scale ranges from 0 to 100. A score under 25 is perceived as a mild complaint, scores between 25 and 50 as clinical significant complaints that need treatment and scores higher than 50 require intensive specialized treatment (Meikle et al., 2012; Rabau et al., 2014). A reduction of 13 points is considered a clinically significant reduction of tinnitus distress, tinnitus severity and the impact of tinnitus on the quality of life (Meikle et al., 2012).

#### Secondary Outcome Measurements

##### Visual analog scale of tinnitus loudness

Secondary outcomes included questionnaire measures. The mean and maximum loudness of the tinnitus during the day were scored on a visual analog scale of tinnitus loudness (VAS_Loudness_), scaled from 0 (absence of tinnitus) to 100 (as loud as possible). Patients indicate the average loudness on a horizontal line. The left end indicating “no tinnitus” and the right end indicating “as loud as you can imagine” (Adamchic et al., 2012).

##### Tinnitus questionnaire

In the tinnitus questionnaire (TQ) the tinnitus related distress is rated on 52 items. The questions are answered on a three-point scale ranging from 0 (“true”), 1 (“partly true”) to 2 (“not true”). Emotional and cognitive distress, intrusiveness, auditory perceptual difficulties, sleep disturbances, and somatic complaints were scored. The total score divides patients in four categories: mild complaints (up to 30 points), moderate complaints (31–46 points), severe complaints (47–59 points), and very severe complaints (60–84 points) (Hallam et al., 1988; Meeus et al., 2007; Hallam, 2008).

##### Hospital anxiety and depression scale

To screen for the presence of signs of depression and/or anxiety the HADS was used (Zigmond and Snaith, 1983; Wilkinson and Barczak, 1988; Spinhoven et al., 1997). This 14-item questionnaire makes use of four answer options for each question. The summation of the subscales gives a signal for the possible occurrence of depression or anxiety in need for specific treatment. A score between 8 and 10 on a subscale indicates “borderline” psychological morbidity and a score of more than 10 indicates a significant “case” of anxiety or depression.

##### Hyperacusis questionnaire

The HQ surveyed the hypersensitivity to sound by use of 14 self-rating items with a four-point answer scale (“no” scoring 0, “yes, a little” scoring 1, yes, quite a lot’ scoring 2, and “yes, a lot” scoring three points). A score of 28 is used as the cut-off for auditive hypersensitivity (Khalfa et al., 2002; Meeus et al., 2010; Fackrell et al., 2015). However, recent research proposed a cut-off HQ score of 16 for classifying hyperacusis (Oishi et al., 2017).

##### Global perceived effect

The global perceived effect scale was used to investigate the sense of improvement each patient experienced after treatment. Ratings consisted of a seven-point transition scale and indicated the subjective opinion on the improvement or deterioration since the start of the treatment (Hudak and Wright, 2000; Kamper et al., 2010). Tinnitus complaints were rated from −3 (much worsened), −2 (worsened), −1 (slightly worsened), 0 (not changed), 1 (slightly improved), 2 (much improved) to 3 (a lot improved).

Psychoacoustic tinnitus analysis was conducted at T_0_, T_1_, and T_2_, consisting of pitch matching, loudness matching, and residual inhibition testing. The tinnitus pitch was determined by means of a forced-choice technique. The patient was asked to choose between two presented pure tones or small band noises (depending on the type of tinnitus) in order to find the best possible match. The stimuli were presented in the contralateral ear. In the case of central tinnitus, the test ear was chosen arbitrarily. The tinnitus loudness matching was performed by comparing the patient’s tinnitus to a pure tone or small band noise of the matched frequency in the ipsilateral ear. The loudness could be changed with an accuracy of 1 dB HL. The intensity of the matched sound is expressed in dB HL and dB SL, with the reference level of dB being the average hearing threshold of a normal-hearing listener and patient’s threshold, respectively. The residual inhibition is the ability of a sound to suppress the patient’s tinnitus, which is measured by presenting a narrow band noise in the ipsilateral ear at an intensity 15 dB louder compared to the matched loudness for one minute. This test results in no change, partial suppression, total suppression, or tinnitus increase (i.e., rebound).

### Statistical Analysis

The current study aimed to (1) assess whether a bimodal therapy for chronic subjective tinnitus consisting of the combination of TRT and EMDR results in a clinically significant different efficacy in comparison with the prevailing bimodal TRT and CBT therapy, and (2) evaluate the effect of the bimodal treatment on the primary (i.e., TFI) and secondary outcome measurements.

Data were analyzed using SPSS statistical software version 25 (SPSS Inc., Chicago, IL, United States). There were three repeated measurements for each individual. To test the first research question, a linear mixed model was constructed, with the treatment group as fixed effect and the interaction between treatment group and time added to the model. The second research question was investigated using a similar linear mixed model, which included time as fixed effect and a random intercept, accounting for the non-independence between the observations taken from the same individual. If the effect of time was significant, a *post hoc* analysis with Bonferroni correction for multiple testing was conducted.

The analyses were carried out using linear mixed model analysis, which is robust against data missing at random. To assess the goodness of fit for the mixed models, we carried out a Levene’s Test for equality of variances. In addition, homoscedasticity was visually inspected using boxplots.

## Results

This randomized controlled trial was conducted aiming to investigate the effect of bimodal treatment TRT/EMDR compared to the effect of bimodal treatment TRT/CBT on the tinnitus outcome measurements. All means and standard deviations at T_0_, T_1_, and T_2_ are shown in Table 2. The estimated group differences, *p* values and effect sizes for primary and secondary outcomes are compared in Table 3.

**TABLE 2:** Primary and secondary outcome measures at baseline, after treatment and 3 months after treatment.

	T_0_ Pre-treatment (baseline)	T_1_ Post-treatment (after 10 therapy sessions)	T_2_ Follow – up (after 3 months)
**Primary outcomes**			
**Tinnitus functional index (SD)**			
Bimodal therapy 1 (TRT/EMDR)	53.88 (18.40)	42.18 (20.67)	37.71 (21.41)
Bimodal therapy 2 (TRT/CBT)	49.60 (21.93)	33.56 (23.15)	34.49 (23.63)

**Secondary outcomes**			

**Tinnitus questionnaire (SD)**			
Bimodal therapy 1 (TRT/EMDR)	39.65 (15.19)	31.26 (17.69)	27.74 (17.10)
Bimodal therapy 2 (TRT/CBT)	41.21 (17.04)	29.40 (18.35)	29.26 (18.55)
**Hyperacusis questionnaire (SD)**			
Bimodal therapy 1 (TRT/EMDR)	22.98 (7.29)	20.22 (7.75)	19.46 (7.81)
Bimodal therapy 2 (TRT/CBT)	22.44 (9.02)	17.93 (9.17)	19.00 (9.16)

**Hospital anxiety and depression scale (SD)**			

**Anxiety**			
Bimodal therapy 1 (TRT/EMDR)	9.39 (4.11)	8.00 (3.37)	7.26 (3.56)
Bimodal therapy 2 (TRT/CBT)	9.49 (4.57)	6.90 (4.36)	6.74 (4.97)
**Depression**			
Bimodal therapy 1 (TRT/EMDR)	7.54 (4.39)	6.24 (3.95)	5.54 (4.14)
Bimodal therapy 2 (TRT/CBT)	7.26 (4.03)	4.86 (4.31)	5.12 (4.40)
**Visual analog scale loudness (SD)**			
Bimodal therapy 1 (TRT/EMDR)	60.49 (23.90)	56.83 (23.75)	52.57 (26.78)
Bimodal therapy 2 (TRT/CBT)	59.55 (21.57)	44.83 (25.72)	46.00 (26.68)

**Global Perceived Effect**			

**Bimodal therapy TRT/EMDR**			
TRT			0.78 (0.76)
TRT + EMDR			1.32 (0.96)
**Bimodal therapy TRT/CBT**			
TRT			0.92 (0.86)
TRT + CBT			1.32 (0.85)

**TABLE 3:** Group differences for primary and secondary outcomes.

	TIME		TREATMENT x TIME	
	T_0_	T_1_	T_0_	T_1_
**Primary outcome**				
Tinnitus functional index	15.11(4.96)***	−0.60(−0.26)	1.06 (0.25)	5.07 (1.58)
**Secondary outcome**				
Tinnitus questionnaire	11.95(5.44)***	0.09 (0.05)	−0.04(−0.01)	3.44 (1.50)
Hyperacusis questionnaire	3.44(3.12)**	−1.11(−1.34)	0.08 (0.05)	1.87 (1.63)
**Hospital anxiety and depression scale**				
Anxiety	2.74(4.64)***	0.11 (0.25)	−0.61(−0.75)	0.63 (1.01)
Depression	2.14(3.68)***	−0.36(−0.81)	−0.14(−0.17)	1.05 (1.73)
Visual analogue scale tinnitus loudness	14.70(4.05)***	−1.08(−0.39)	−6.81(−1.35)	5.34 (1.40)

Estimates (*t*-values) for the effect of time and treatment group on the primary and secondary outcome measures. Reference category is T_2_ and CBT. ** *P* < 0.01; *** *P* < 0.001.

**FIGURE 3:**
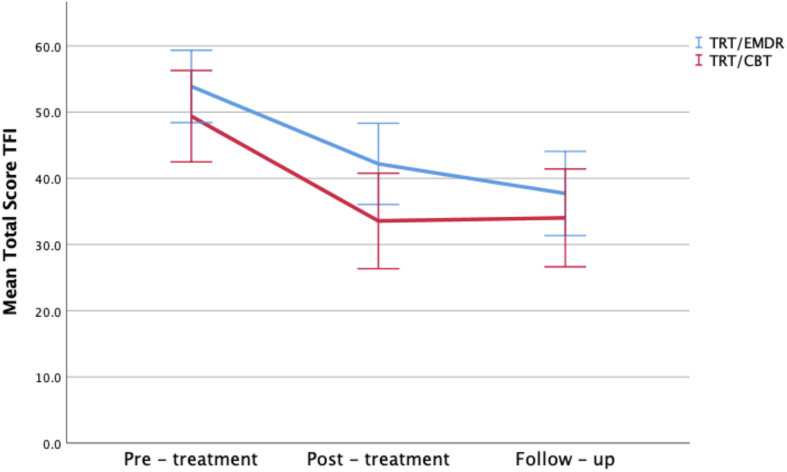
Total TFI scores by therapy group and time period. Error bars representing 95% CI interval.

The change on the TFI total score over time was statistically significant in both bimodal therapies (*p* < 0.001), with a mean improvement of 15.7 points from T_0_ to T_2_ (respectively a mean decrease of 15.1 in TRT/CBT; *p* < 0.001 vs. 16.2 in TRT/EMDR; *p* < 0.001). This effect was not significantly different between the two treatment groups (*p* > 0.05). *Post hoc* analysis showed no significant changes between T_1_ and T_2_. In other words, the level of improvement remained stable after 3 months subsequent to completion of the therapy (Figure 3).

**FIGURE 4:**
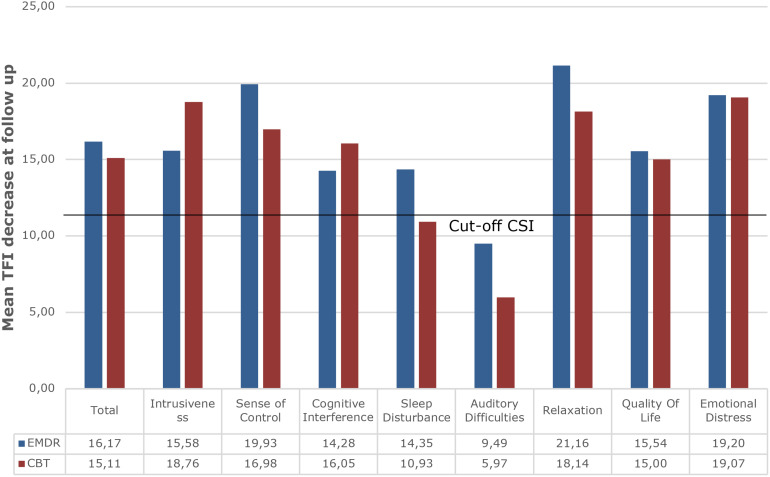
Overview of mean decrease at follow up on all TFI subscales for both treatment groups indicating the cut-off of a decrease of 13 points as marker for Clinically Significant Improvement (CSI). TRT/EMDR treatment group presented in dark blue bars and TRT/CBT treatment group in red bars.

Figure 4 depicts an overview of all TFI – subscales. Clinically significant improvement was reached on every subscale in both treatment groups, except for the subscale Sleep Disturbances after TRT/CBT and the subscale Auditory Difficulties after TRT/CBT and TRT/EMDR.

**FIGURE 5:**
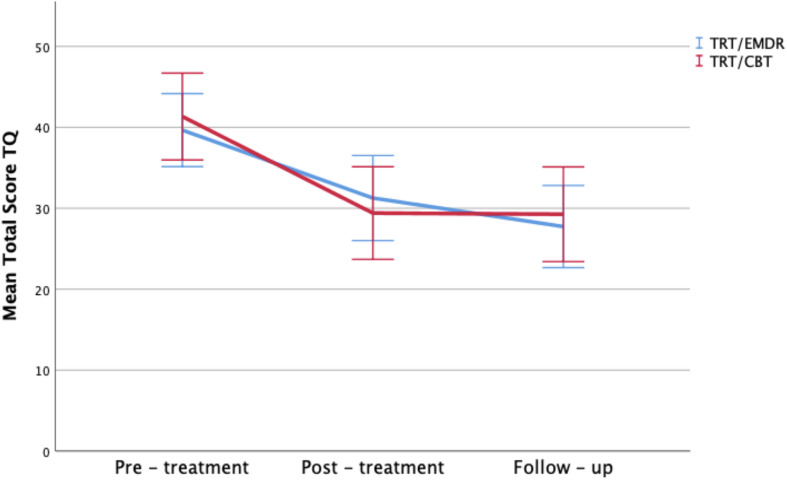
Total TQ scores by therapy group and time period. Error bars representing 95% CI interval.

**FIGURE 6:**
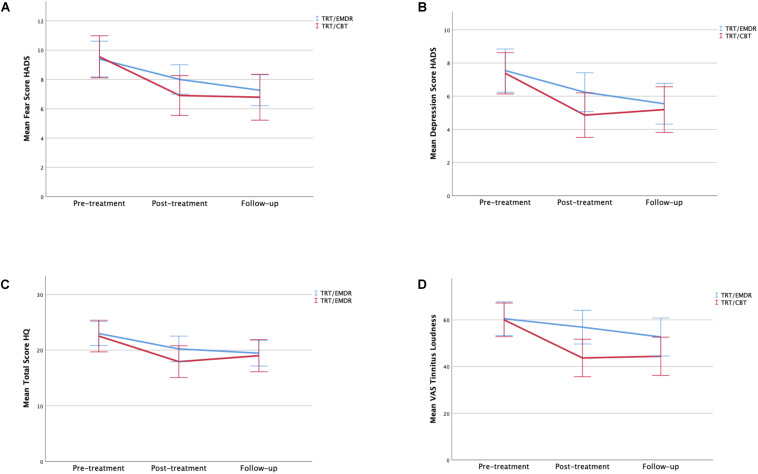
**(A)** Mean of total fear scores by therapy group and time period. **(B)** Mean of total depression scores by therapy group and time period. **(C)** Mean of total HQ scores by therapy group and time period. **(D)** Mean of total VAS scores of tinnitus loudness by therapy group and time period. Error bars representing 95% CI interval.

With regards to the secondary outcomes, the TQ total score (Figure 5), HADS anxiety score, HADS depression score, HQ score, and VAS of tinnitus loudness also improved significantly over the three visits (*p* < 0.001) (Figure 6). The tinnitus frequency also decreased over the three assessments, and this trend was significant for the right ear (*p* < 0.05).

When comparing the effects over time between the two treatment groups, there was a significant effect on the TFI-subscale Relaxation between the TRT/CBT group and the TRT/EMDR group (*p* < 0.05). *Post hoc* comparisons revealed that the TRT/CBT group did not show a significant change in the follow-up period (*p* > 0.05), whereas the TRT/EMDR group did (*p* < 0.05). A mean decrease of 20.3 points in the TRT/CBT group in comparison to a mean decrease of 11.7 in the TRT/EMDR group was found at T_1_. Between T_1_ and T_2_, the TRT/CBT group showed a slight increase of 2.0 points and the TRT/EMDR group showed a significant decrease of 9.5 points. Second, a significant difference in improvement (*p* < 0.05) was indicated on the TQ-subscale Somatic complaints. The TRT/CBT group improved significantly from T_0_ to T_1_ (*p* < 0. 01) (mean = 0.7), whereas the TRT/EMDR group improved significantly from T_1_ to T_2_ (*p* < 0.05) (mean = 0.5). Subsequently, on the VAS for mean tinnitus loudness, a significant difference in decrease was detected in favor of the TRT/CBT therapy between pre-therapy and post-therapy (*p* < 0.01), with this group showing a mean improvement of 16 points (*p* < 0.001) in comparison to a mean improvement of 4 points in the TRT/EMDR group. The TRT/EMDR group showed a significant improvement over time (*p* < 0.05), but the *post hoc* comparisons between the time points were not significant. No significant differences were found on the other primary or secondary outcome measurements.

Finally, the analysis of the GPE shows a positive effect of the bimodal therapy approach (*p* < 0.001). The GPE does not differ significantly between the two treatment groups.

## Discussion

In the present study the effect of two bimodal therapies in the treatment of chronic, subjective tinnitus was compared. TRT was included in the treatment protocol to create a baseline therapy level. Starting from the same amount of psychoeducation – due to the TRT therapy – in every therapy group a comparison could be made between EMDR and CBT. The implementation of TRT also reduced the risk of bias as patients were given treatment and thus prevented the risk of drop-out.

The primary interest was to investigate whether EMDR could be implemented in the treatment of tinnitus. The most interesting finding was that TRT/EMDR showed clinically significant decrease in tinnitus complaints and associated life domains in comparison to TRT/CBT in the current study protocol. There was no inferiority found for bimodal therapy TRT/EMDR compared to bimodal therapy TRT/CBT. Both treatment arms resulted in a clinically significant improvement of the TFI total score (i.e., a decrease of ≥13 points) (Meikle et al., 2012). Moreover, the participants also showed significantly decreased scores on the TQ, HQ, HADS and VAS for tinnitus loudness. The present study has shown that both therapeutic trajectories offer clinically significant improvement for patients suffering from tinnitus and hyperacusis. Previous research has shown that CBT for hyperacusis shows promising results (Jüris et al., 2014). This RCT highlights the positive effects of TRT/CBT and TRT/EMDR on tinnitus complaints and the high prevalent comorbid complaints such as hypersensitivity to sounds, anxiety and depression.

The power of the current study design was calculated to detect a difference in effect between the two treatments. This RCT, with 43 and 46 persons in the two treatment groups, offers 80% power to detect a difference in effect (i.e., decrease of ≥13 points on the TFI) of 0.6 standard deviations (Cohen’s D) between both groups at a significance level of 0.05. Assuming that the standard deviation within the treatment groups is around 20 (Table 3), this would imply a difference in effect of 12 units between both treatments. Increasing the sample size to 400 individuals per group would have a power of 80% to detect a difference of 0.4 standard deviations, which is equivalent to a difference in 8 units between both groups.

Therefore, the current study is only able to detect large differences in effect between the two treatments. The non-significance of the interaction between treatment and time may be attributable to a lack of power in the current study, that does not allow to detect subtle differences in effect. In this context we do want to point out the strong significant therapeutic effect that was detected in both therapy arms. Therefore, it would have been challenging to significantly transcend the obtained improvement by one or the other bimodal treatment.

Notably, previous research concerning the comparison between EMDR and CBT demonstrates similar outcome with no significant differences in the treatment of PTSD (Van Etten and Taylor, 1998; Seidler and Wagner, 2006; de Roos et al., 2011; Ho and Lee, 2012; Santarnecchi et al., 2019), panic (Faretta, 2013), and obsessive compulsive disorder (Marsden et al., 2018). Based on the AIP-model, EMDR may promote plasticity of the brain causing adjustment of neural networks and the development of new adaptive networks. This theory supports the findings of Jastreboff and Jastreboff (2000) indicating that retraining the brain to achieve habituation of the tinnitus-induced reactions and tinnitus perception is due to the plasticity of the neural networks. Memory mechanisms are known to have a major impact on the persistence of the awareness of the phantom percept and the associated distress (De Ridder et al., 2011). Aiming at reorganizing different networks containing the hippocampus, parahippocampus, anterior insula, amygdala, subgenual and dorsal anterior cingulate cortex, parietal cortex, posterior cingulate cortex and precuneus, and prefrontal cortex is therefore necessary (De Ridder et al., 2011). The decrease in frontal activation and the increase of limbic processing as a consequence of bilateral stimulation could result in effective information processing causing the tinnitus percept to change (Herkt et al., 2014; Boukezzi et al., 2017).

Furthermore, our results are consistent with the findings of the pilot study of Rikkert et al. (2018) reporting significant reduction on the TFI after EMDR-treatment. The other exploratory study on EMDR and tinnitus (Phillips et al., 2019) also showed an improvement after treatment with EMDR with a mean decrease of 20 points on the THI which remained stable at the 6-month follow up. Similar to our study, Rikkert et al. conducted a 3-month follow up, also concluding a stable treatment effect over time.

Different theories have been considered on the working mechanisms of EMDR such as the working memory account, classic conditioning, physiological changes associated with the orienting response, REM sleep, changes in interhemispheric connectivity, neural integration and thalamic binding, and structural and functional brain changes (Landin-Romero et al., 2018). Still no unanimity has been reached on the most explanatory model. There is abundant room for further progress in determining which components of effective treatments tackle the reorganization of the responsible neural correlates, but these matters are beyond the scope of this study. No substantial evidence was found to explain the significant intergroup differences on the TFI-subscale Relaxation, TQ-subscale Somatic complaints and VAS_Loudness_ after treatment that were found in this study. We speculate that different therapeutic interventions integrated in a different way over time and reflected the progress during the treatment course.

The current randomized controlled trial holds several strengths. Participants were randomly assigned to one of the two treatment conditions by using a stratification paradigm according to TFI grade. The heterogeneous group of patients indicate generalizability for both treatments. A realistic presentation of the current tinnitus population with the occurrence of sleep difficulties, anxiety and depressive symptoms was investigated. More than 50% of the participants reported experiencing tinnitus for more than 5 years and tried several therapies in the past without success. This clinical study has included an appropriate sample size. Only two patients were unable to complete the therapeutic trajectory. The drop-out rate was very low indicating that patients were motivated, and a positive therapeutic alliance was built. The study design enhanced the compliance with the treatment. A double-blind design was used, for the participants, for the investigator and the TRT-therapist. Only the psychotherapist conducting the CBT and EMDR treatments was unblinded. All patients received the same amount of contact hours and were treated by the same therapists. Therefore, there was no bias on duration of the therapy and therapist effects could be controlled. However, this could also be considered a shortcoming as a “crossed therapist design” could influence outcome if an allegiance toward one therapy exists (Falkenström et al., 2013). This possible bias was assessed in this study and no differential psychotherapist allegiance was found.

The GPE served as assessment to achieve data about the level of improvement after treatment. Evaluations of the patients on the GPE scale provided valuable information about the capital gain of the treatment providing insights into the experiences of the tinnitus sufferers. More than 80% of the patients reported improvement after the TRT/EMDR treatment and more than 84% described reduction of tinnitus complaints after the TRT/CBT intervention and expressed feeling better to a lot better. Previous research has emphasized the importance of tailored treatment and the uniqueness of every tinnitus patient (Shi et al., 2014; Van de Heyning et al., 2015). Therefore, we stress the need to listen carefully to each individual patient and focus on their needs.

The design of the study had some constraints. The implementation of an active control group was indicated to investigate the efficacy of EMDR compared to the known effective treatment. Unfortunately, placebo effects are difficult to rule out when using an active control design (Boot et al., 2013). Even though there are risks associated with an active control group, it would have been unethical to abstain these patients with bothersome tinnitus from the actual treatment. A placebo treatment could be considered, but one might question if the presence and attention given by a health professional could enhance hope, expectations and reassurance, as a waiting list condition could also induce the same effects. Investigating psychotherapy comes with these particular challenges (Boot et al., 2013). The prior concern should be the well-being of the patient and the focus on mental and physical resilience.

The effectiveness of both therapies may be due to the common factors present in EMDR and CBT. Lambert (2016) state that different areas can influence client outcome, namely specific therapy techniques, expectancy effects, extra therapeutic factors, and common factors. Studies on psychotherapeutic interventions have demonstrated that common factors such as empathy, congruence, unconditional positive regard, and the therapeutic alliance correlate more highly with client outcome than specialized treatments (Lambert, 2016; Cuijpers et al., 2019).

The current RCT reports effectivity for two different effective bimodal therapies. Remarkably it seems that effectivity may find the basis in the specific treatment elements and more importantly in common factors such as the therapeutic alliance. Moreover, to develop a full picture of the effectivity and implementation of EMDR in the treatment of chronic, subjective tinnitus, additional studies are required. In future investigations, it might be possible to use a control group that can take several variables into account such as specific therapy techniques, expectancy effects, and common factors, which all can have an effect on the client outcome. Additionally, recent attention has been given to the relations between tinnitus distress and personality traits and consequently the inevitable impact on treatment (Simões et al., 2019). Future research may focus on these elements as possible key factors for effectivity in the treatment of tinnitus.

## Conclusion

In conclusion, these results indicate that there might be a different effect or influence on certain life domains caused by either EMDR or CBT resulting in better coping strategies, different associations and reactions toward the tinnitus. However, a clear superiority of TRT/EMDR in the treatment of tinnitus could not be identified in this analysis. Evidence was found that both therapies prove to lead to significant improvement of the tinnitus perception and the tinnitus distress.

## Data Availability Statement

The raw data supporting the conclusions of this article will be made available by the authors, without undue reservation.

## Ethics Statement

The studies involving human participants were reviewed and approved by the Ethical Committee of the Antwerp University Hospital on October 17, 2016 with protocol number EC UZA 16/35/360. The patients/participants provided their written informed consent to participate in this study.

## Author Contributions

TL, LJ, FD, EF, NV, PV, and AG conceived and designed the study. TL drafted the manuscript. TL, LJ, NV, EC, and EF performed the treatments, data collection, and analysis. PV, FD, EF, NV, EC, MD, VT, VV, LJ, and AG critically reviewed the manuscript. All authors read and approved the final manuscript.

## Conflict of Interest

TL and NV were employed by the company BVBA Hoorzorg Van Looveren. The remaining authors declare that the research was conducted in the absence of any commercial or financial relationships that could be construed as a potential conflict of interest.

## Appendix 1 summary of treatment protocol TRT/CBT

### Bimodal Therapy TRT/CBT

#### Psychological Intake

• Performed by a clinical psychologist (60 min), a certified psychotherapist
• Patient history
• Assessment of complaints associated with tinnitus and comorbidities
• Questions about important life events
• Current coping strategies
• Questions about previous treatment

### TRT

Performed by a clinical audiologist and certified TRT-therapist (accompanied by a PowerPoint presentation)

#### Session 1 (60 min)

• Assessment of tinnitus complaints
• Assessment of present audiologic support (hearing aids if necessary, sound enrichment) if present
• Start tinnitus counseling: an explanation of the anatomy and physiology of the normal and impaired auditory system

#### Session 2 (60 min)

• Continuation of the counseling: role of the central auditory system and higher cortical processes, sensory contrast, selective perception of sensory information

#### Session 3 (60 min)

• Continuation of the counseling: an explanation of the neurophysiological model of tinnitus by Jastreboff, illustrate the importance of the activation of subcortical, limbic and automatic nervous systems in tinnitus

#### Session 4 (60 min)

• Explanation of the importance of sound enrichment, startup of sound generators (bilateral) and clarification about the use

#### Session 5 (60 min)

• Evaluation of the hearing aids, sound generator, sound enrichment^∗^
• Recapitulation of the tinnitus – specific psychoeducation
• Handouts of the TRT counseling with personal notes and tips and tricks to cope, information about stress reduction, relaxation exercises, sleep hygiene, concentration and hearing tips

### CBT

Performed by a clinical psychologist, a psychotherapist in cognitive behavioral therapy (accompanied by a PowerPoint presentation; based on the CBT treatment outline by Dr. Aazh from the Tinnitus and hyperacusis master class in 2013, Birkbeck College, London)

#### Session 1 (60 min)

• Education about the auditory system and psychoeducation (brief, only if necessary)
• Introduction of CBT and assess the motivation and commitment toward the therapy
• Counseling and identification of thoughts, emotions, and behavior to the tinnitus sound using the ABC-model
• Create time and space to focus on personal concerns and give reassurance (every session)

#### Session 2 (60 min)

• Counseling and identification of negative automatic thoughts and core belief
• Educating about common distortions in thinking: all or nothing thinking, overgeneralizing, mental filter, disqualifying the positive, jumping to conclusions, mind reading/fortune-telling, catastrophizing and minimization, emotional reasoning, should/must statement, labeling, personalization

#### Session 3 (60 min)

• Counseling about challenging unhelpful thoughts, errors of judgment and creating counterstatements
• Cognitive restructuring

#### Session 4 (60 min)

• Evaluate the experiences of the patient as a consequence of helpful thoughts
• Behavioral desensitization and graded exposure: specified to be integrated into the patient’s life

#### Session 5 (60 min)

• Evaluate the therapy process and progress^∗^
• Focus on positive changes and possible future perspectives
• Identify challenges and helpful coping strategies, depending on the patients’ needs focus on elements of mindfulness, acceptance and commitment therapy, relaxation techniques

^∗^Follow – up was organized if necessary (telephone contact, supporting e-mail contact or an extra appointment for fitting or adapting the hearing aids).

## Appendix 2 Summary of Treatment Protocol TRT/EMDR

### Bimodal Therapy TRT/EMDR

#### Psychological Intake

Identical to bimodal therapy TRT/EMDR

### TRT

Identical to bimodal therapy TRT/EMDR

### EMDR

Performed by a clinical psychologist, a Europe Practitioner in Eye Movement Desensitization and Reprocessing, phases were adapted according to the EMDR protocol of Shapiro and the pace and needs of the patient

#### Session 1 (60 min)

• Phase 1: Client history: identifying the negative thoughts, sensations, and experiences associated with the tinnitus complaints
• Phase 2: Preparation phase: assessment of the stability of the patient, (if the patient was not stable, stabilization techniques were applied), installation of the calm or safe place (use of slow bilateral stimuli), secure a stop sign for when the patient wanted to stop the desensitization process
• Create time and space to focus on personal concerns and give reassurance (every session)
• Phase 3: Assessment phase: the following elements were made salient: target issue, memory, event or symptom associated with the tinnitus, target image, negative cognition (NC), positive cognition (PC), Validity of Cognition (VoC) on a scale of 1 - 7, emotions, location of bodily sensation, Subjective Units of Distress (SUD) on a scale of 0 – 10

#### Session 2 (60 min)

• Reevaluation of the previous session Recapitulate phase 3
• Phase 4: Desensitization phase: focus on the target image, negative cognition and bodily sensation, set of bilateral stimuli = BLS (visual or tactile) as fast as the patient can tolerate, after each set the therapist asked the question “What do you get now?, What are you noticing?”, if the patient reports new material “Go with that.”, after the patient reported two consecutive times positive material (=desensitization), phase 5-6-7 was started
• If phase 4 was not completed at the end of the session, the therapist ensured stabilization before leaving the private practice according to the standard protocol; discussion of what can appear after the session

#### Session 3 (60 min)

• Reevaluation of the previous session
• Recapitulate phase 3 and phase 4: perform BLS until desensitization was reached
• Phase 5: Installation phase: check PC “Do the words PC still fit, or would another positive statement be more suitable?”, and VoC “Think about the original incident and the words PC. How true do they feel now (1-7)?”
• The patient was asked to focus on the target incident and the PC, followed by completion of sets of BLS until there was no change
• Phase 6: Body scan: the patient was asked to mentally scan the entire body and report this to the therapist
• Phase 7: Closure: check of feelings and sensations of the patient; identification of the gain of the session and discussion of what can appear after the session

#### Session 4 (60 min)

• Phase 8: Reevaluation of the previous session: ^∗^the patient assesses the results and experiences since the previous session
• Recurrence of phase 3 – phase 7 when other targets were associated with the tinnitus complaints

#### Session 5 (60 min)

• Phase 8: Reevaluation of the previous session^∗^
• Recurrence of phase 3 – phase 7 when other targets were associated with the tinnitus complaints

^∗^Follow – up was organized if necessary (telephone contact, supporting e-mail contact or an extra appointment for fitting or adapting the hearing aids).
